# Case Report: Four cases of SARS-CoV-2-associated Guillain-Barré Syndrome with SARS-CoV-2-positive cerebrospinal fluid detected by metagenomic next-generation sequencing: a retrospective case series from China

**DOI:** 10.3389/fimmu.2023.1258579

**Published:** 2023-08-28

**Authors:** Yalin Guan, Changshen Yu, Yunhan Fei, Qiushi Wang, Pan Wang, Wenchao Zuo, Hao Wu, Xuemei Qi, Qiyun Shi

**Affiliations:** ^1^ Department of Neurology, Tianjin Huanhu Hospital, Tianjin, China; ^2^ Department of Emergency, Tianjin Huanhu Hospital, Tianjin, China; ^3^ Infection Business Unit, Tianjin Novogene Med LAB Co., Ltd., Tianjin, China; ^4^ Infection Business Unit, Novogene Co., Ltd., Beijing, China; ^5^ Department of Neurology, The second hospital of tianjin medical university, Tianjin, China

**Keywords:** SARS-CoV-2, Guillain-Barré Syndrome, metagenomic next-generation sequencing, cerebrospinal fluid, COVID-19

## Abstract

Severe acute respiratory syndrome coronavirus 2 (SARS-CoV-2) is often absent or at low levels in the cerebrospinal fluid (CSF) of patients with previous SARS-CoV-2-associated Guillain-Barré syndrome (GBS). This has led to speculation that SARS-CoV-2-associated GBS is more likely mediated by post-infectious immunity or a parainfection. This understanding has influenced the development of treatment regimens for SARS-CoV-2-associated GBS. This paper reports our experience with four Chinese patients with SARS-CoV-2-associated GBS who tested positive for SARS-CoV-2 RNA in the CSF. They developed symptoms of peripheral nerve damage 4–15 days after fever and confirmed SARS-CoV-2 infection, all of whom presented with progressive weakness of both lower limbs; three with autonomic nerve function impairment such as constipation and urination disorder; and one with polycranial neuritis and Miller–Fisher syndrome. Three patients were tested for anti-ganglioside antibodies, and one tested positive for GD1a-IgG. Four patients recovered well after treatment with anti-viral drugs combined with intravenous immunoglobulin. The present results showed that SARS-CoV-2 RNA can be detected via mNGS in the CSF of some patients with SARS-CoV-2-associated GBS, suggesting that SARS-CoV-2-associated GBS may have multiple pathogeneses.

## 1 Introduction

Since the beginning of 2020, coronavirus disease (COVID-19) caused by severe acute respiratory syndrome coronavirus type 2 (SARS-CoV-2) has spread rapidly around the world, profoundly impacting human health, lifestyle, economy, politics, and even the world pattern. SARS-CoV-2 mainly affects the respiratory system, causing fever, cough, and dyspnea (1, 2) and can also cause a variety of SARS-CoV-2-related nervous system inflammatory diseases through direct infection, immune-mediated inflammatory injury, SARS-CoV-2-related cytokine storm, and autoimmune mechanisms, among which Guillain-Barré Syndrome (GBS) is one of the most common types (3, 4).

The most common clinical manifestations of SARS-CoV-2-associated GBS are lower limb weakness and paresthesia (5, 6), but almost all subtypes and variants of GBS have been reported. The incidence of respiratory failure in SARS-CoV-2-associated GBS is 18.2–38% (5–7), and the mortality rate is 4.5–5.8% (5, 8). Reports of cases of SARS-CoV-2-associated GBS with SARS-CoV-2 RNA positive in cerebrospinal fluid (CSF) are rare, and it is speculated that SARS-CoV-2-associated GBS is unlikely to be related to direct neurological invasion of the virus (9, 10). For this reason, most patients with SARS-CoV-2-associated GBS are treated with intravenous immunoglobulin (IVIg) or plasma exchange (5), and reports of combined antiviral therapy are rare.

In this paper, we report our experience with four Chinese patients with SARS-CoV-2-associated GBS with SARS-CoV-2-positive RNA in the CSF. All patients recovered well after treatment with IVIg in combination with azvudine, an anti-SARS-CoV-2 drug. Our report may shed light on the possible pathogenesis of SARS-CoV-2-associated GBS.

## 2 Case description

Four patients were admitted to the neurology department of Tianjin Huanhu Hospital between Jan 2, 2023, and Jan 11, 2023. The characteristics, image examinations and laboratory examination items of the four patients are listed in **Tables 1**, **2**. Metagenomics next-generation sequencing method is described in the **Supplementary Material**. The details for each patient are provided below.

**Table 1:** Characteristics and image examinations of four cases.

Items	Patient 1	Patient 2	Patient 3	Patient 4
Medical history	History of hypertension for 1 year; hepatitis B virus carriers, not treated	History of hypertension for 10 years	Ovarian cyst resection 3 months ago	Partial resection of right lung due to bronchiectasis more than 40 years ago; Have “penicillin, streptomycin, cephalosporin, roxithromycin” and other drug allergy
Time of last COVID-19 vaccination	2022/12/6	2022/5/9	2022/1/7	2022/7/20
Onset time of peripheral nerve symptoms	2022/12/26	2023/1/2	2022/12/30	2022/12/23
Onset of symptoms according to COVID-19 onset time (days)	13	15	14	4
Brain MRI	Normal	Normal	FLAIR high signal in bilateral frontal, temporal and parietal cortex; linear enhancement in some sulci	Normal
Electrophysiological examination	Peripheral neurogenic damage of limbs (sensory and motor fibers were involved), abnormal F wave of limbs	Peripheral neurogenic damage of limbs (sensory and motor fibers were involved), and the occurrence rate of F wave of both upper limbs and left lower limb was 0%	Abnormal motor evoked potential of both lower limbs	The amplitude of motor nerve conduction of left common peroneal nerve decreased, and the occurrence rate of F wave of left upper limb was low
Lung CT	Rope shadow of right lower lobe	Bilateral pulmonary interstitial changes, pulmonary infection	Double lower lung inflammation	Pulmonary infection, bronchiectasis
Classification according to the Brighton criteria	1	1	2	2

**Table 2:** Laboratory examination results of four cases.

Specimen type	Test items (units) (Normal range)	Patient 1		Patient 2		Patient 3		Patient 4	
		Initial examination	Re-examination	Initial examination	Re-examination	Initial examination	Re-examination	Initial examination	Re-examination
Blood	WBC count (10^9^/L)	7.03	3.88	6.26	5.44	7.58	8.48	7.39	3.38
	lymphocyte count (10^9^/L)	2.56	0.74↓	1.82	2.03	1.3	2.54	1.6	1.13
	PCT (ng/ml) (0-0.046)	0.053↑	Not checked	<0.022	0.042	<0.022	0.037	0.029	0.059↑
	Ferritin (ng/ml) (13-150)	411.70↑	Not checked	161.9	Not checked	75.31	Not checked	128.8	189.7↑
	IgG (mg/L) (7-16)	11.4	30.6↑	17.6↑	32.2↑	15.4	29.7↑	15.7	31.8↑
	IgA (mg/L) (0.7-4)	1.88	1.52	2.89	3.18	1.63	1.43	3.58	3.27
	IgM (mg/L) (0.4-2.3)	0.899	0.794	0.611	0.733	0.592	0.616	0.894	0.764
	SARS-CoV-2 sequence number	2	0	171	0	685	2	0	Not checked
	SARS-CoV-2 specific antibody	IgM (+), IgG (+)	IgG (+)	IgG (+)	IgG (+)	IgG (+)	IgG (+)	IgG (+)	IgG (+)
CSF	Pressure (mmH_2_O)	160	117	165	175	166	142	140	Not checked
	WBC (10^6/L)	8	2	6	8	6	6	2	Not checked
	Protein (g/L)	1.06↑	0.72↑	1.93↑	1.69↑	0.39	0.27	0.37	Not checked
	protein-cell separation	Yes	Yes	Yes	Yes	No	No	No	Not checked
	IgG (mg/L) (0.1-34)	120↑	280↑	343↑	488↑	23.3	34.2↑	32.9	Not checked
	IgA (mg/L) (0.1-5)	10.5↑	9.08↑	50↑	26.8↑	1.4	6.37↑	4.28	Not checked
	IgM (mg/L) (0-1.3)	3.55↑	2.21↑	9.57↑	3.85↑	0.157	0.219	0.289	Not checked
	IgG ratio (0.051-0.183)	0.13	0.50↑	0.30↑	0.49↑	0.13	0.31↑	0.20↑	Not checked
	SARS-CoV-2 sequence number	446	0	700	0	4	0	371	Not checked
	SARS-CoV-2 specific antibody	IgG (+)	IgG (+)	IgG (+)	IgG (+)	IgG (+)	Negative	Negative	Not checked
	Autoimmune encephalitis, paraneoplastic and central nervous demyelinating antibodies	Negative	Not checked	CSF MOG-IgG 1:1	CNS demyelinating (-), others not checked	Negative	AE (-), others not checked	Not checked	Not checked
Blood and CSF	Ganglioside antibody	Negative	Negative	Negative	Negative	Blood GD1a-IgG (+)	Negative	Not checked	Not checked
Blood and CSF	Oligoclonal bands	Negative	Negative	Symmetric OB	Negative	Negative	Negative	Not checked	Not checked

MOG, myelin oligodendrocyte glycoprotein; AE, autoimmune encephalomyelitis; PCT, procalcitonin.

↑ indicates that the value is higher than the normal range, and ↓ indicates that the value is lower than the normal range.

### 2.1 Case 1

A 33-year-old male, had fever and was SARS-CoV-2 antigen-positive 13 days prior to the onset of GBS. The patient was admitted to the hospital on the 8th day after onset. Physical examination on admission revealed conscious, dysarthria, impaired bilateral pharyngeal reflex, bilateral peripheral facial paralysis, limitation of abduction of both eyes, nystagmus when looking to the left, diplopia when looking to the left and righ, grade 4 muscle strength of both upper limbs, grade 3 muscle strength of both lower limbs, normal bilateral sensation, ataxia of the left limbs, impaired tendon reflex of both upper limbs, the absence of tendon reflex of both lower limbs. The patient was administered mecobalamin, neurotropin, pregabalin, and betahistine, after which the patient’s back pain was relieved and dizziness aggravated; however, other symptoms were not relieved. On the 10th day of onset, azvudine was added based on metagenomic next-generation sequencing (mNGS) results. On the 11th day of onset, the patient experienced additional relief from dysphagia and numbness of the limbs. Physical examination revealed that the patient had no nystagmus, no diplopia when looking to the right, and diplopia was relieved when looking to the left. On the 13th day of onset, the patient and his family agreed to receive IVIg after consultation, and the physician added 400 mg/kg of human immunoglobulin. On the 18th day after onset, the patient still had bilateral facial paralysis, bulging cheeks stronger than before, no air leakage, independent walking, unsteady gait, and no induced tendon reflex in either lower limb.

### 2.2 Case 2

A 64-year-old female, presented with fever and was positive for SARS-CoV-2 antigen 15 days prior to this episode. On the first day of onset, the patient experienced weakness and numbness in both lower limbs during activities, walking slightly clumsily, completing housework, and experiencing laborious defecation. On the 2nd day of onset, the patient could not walk independently, and on the 3rd day of onset, the patient could not stand and had numbness in both upper limb. She went to the local health center, where the village doctor thought her symptoms might be caused by an infection and administered moxifloxacin empirically, but her condition progressed. On the 4th day of onset, the patient experienced urinary incontinence and was hospitalized. The muscle strength of both upper limbs was grade 5 and that of both lower limbs was grade 3. The patient had perianal hypoesthesia, hyperalgesia in both feet, absence of tendon reflexes in both lower limbs, and negative pathological signs bilaterally. After treatment with mecobalamin and neurotropin, the patient’s condition continued to worsen. On the 5th day of onset, SARS-Cov-2 was detected by mNGS in blood and CSF. On the 6th day of onset, the patient had grade 2 strength of both lower limbs and was administered azvudine and IVIg on the same day, and the patient’s condition gradually improved. On the 16th day of onset, the patient was able to walk with assistance, numbness in the hands was lessened, urinary incontinence disappeared, and the patient was discharged.

### 2.3 Case 3

A 40-year-old female, developed a fever 14 days prior to this episode and was confirmed to be infected with SARS-CoV-2 by nucleic acid testing of throat swabs. After onset, the main symptom was weakness in both lower limbs, which gradually worsened to the inability to walk within 4 days, accompanied by dizziness and difficulty in urinating and defecating. The patient was admitted to the hospital on the 4th day after onset. Physical examination on admission revealed grade 3 muscle strength of both lower limbs, decreased muscle tone and tendon reflex of both lower limbs, and negative pathological signs bilaterally. Upon admission, the patient was administered ganciclovir, mecobalamin and neurotropin. The patient’s condition gradually progressed to grade 2 muscle strength in both lower limbs. On the 6th day after onset, azvudine was administered according to the results of mNGS. One day after treatment, the weakness in both lower limbs was slightly relieved. On the 7th day of onset, IVIg was added, the patient’s urinary function recovered, and the catheter was removed. On the 13th day of onset, the weakness in both lower limbs was significantly relieved, the muscle strength in both lower limb was grade 4, and the difficulty in defecation was relieved.

### 2.4 Case 4

A 64-year-old female, presented with fever, headache, cough, sore throat, and SARS-CoV-2 antigen positivity four days prior to this episode. After onset, the patient experienced gradually worsening weakness in both lower limbs and skin pain in the chest and abdomen. On the 19th day of onset, the patient was completely unable to move both lower limbs and was admitted to the hospital. Physical examination on admission showed grade 0 muscle strength of both lower limbs, decreased muscle tension and tendon reflex of both lower limbs, superficial hypoesthesia of both lower limbs, and negative pathological signs bilaterally. On the 20th day of onset, 371 sequences for SARS-Cov-2 were detected by mNGS in CSF. Azvudine combined with IVIg was administered on the 21st day of onset, after which the patient’s condition gradually improved. On the 27th day of onset, muscle strength in both lower limbs was grade 4, and the patient was able to walk with assistance.

**Figure 1:**
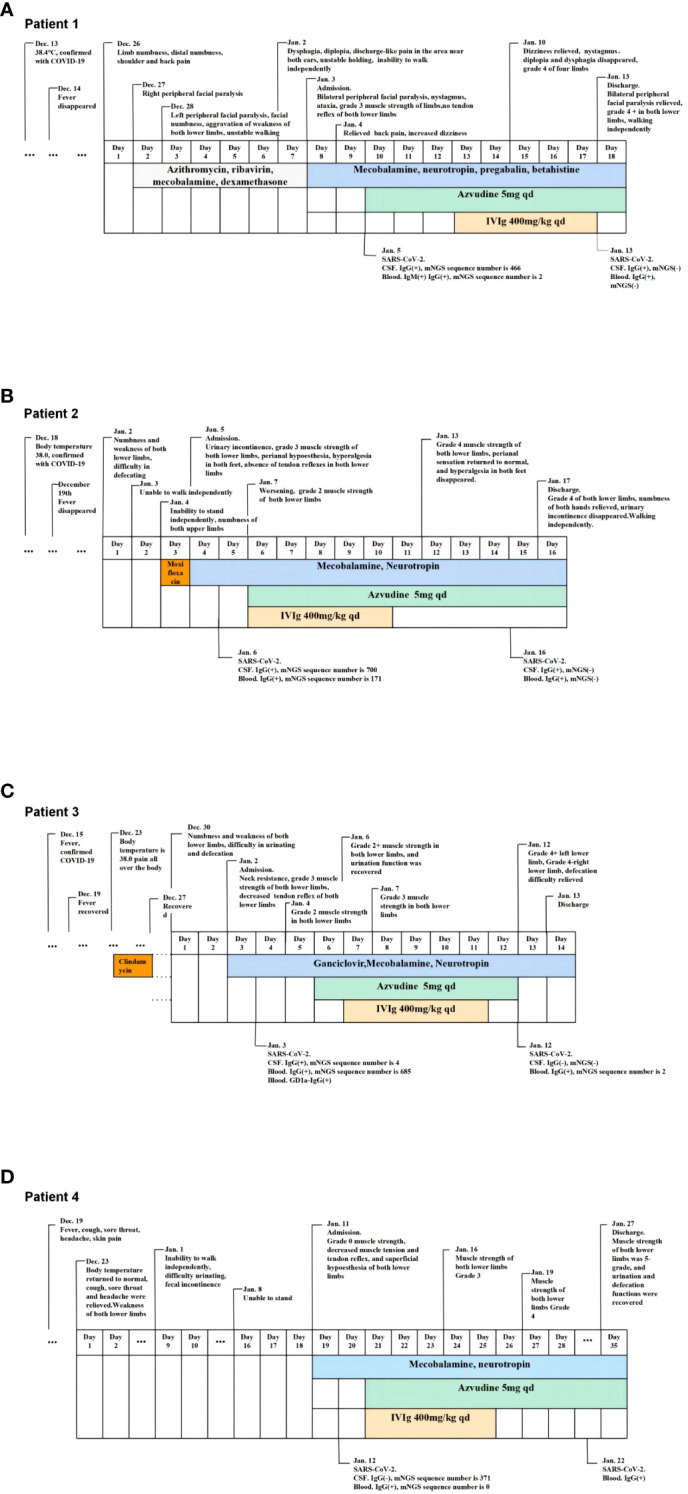
The timeslines of Case1-4. **(A)** The timeline of case 1. **(B)** The timeline of case 2. **(C)** The timeline of case 3. **(D)** The timeline of case 4.

The timelines of Cases 1-4 are shown in **Figure 1**.

Patients 1, 2, and 3 were tested for autoimmune encephalitis, central nervous system demyelination, ganglioside antibodies, Ranvier node antibodies, and paraneoplastic antibodies in the blood and CSF. Patient 4 refused antibody screening. Patients 1 and 2 tested negative for all the aforementioned antibodies; Patient 3 tested positive for GD1a-IgG and negative for the other antibodies. The exact antibody types tested are described in the **Supplementary Material**.

## 3 Discussion

In this paper, we report our experience with four SARS-CoV-2-associated GBS cases in China, in which SARS-CoV-2 RNA was found in the CSF. The age distribution of the patients was 33–64 years, with a male:female ratio of 1:3. All four patients had a history of other diseases. SARS-CoV-2 infection was confirmed by nucleic acid or SARS-CoV-2 antigen detection from throat swabs taken from all patients after fever and other symptoms. At the time of diagnosis of SARS-CoV-2 infection, only one patient had respiratory symptoms such as cough, and this patient had a history of bronchiectasis and had undergone lobectomy. One patient developed fever with generalized pain, and the other two patients only had fever. Four patients developed neurological symptoms 4–15 days after COVID-19 symptoms and confirmed SARS-CoV-2 infection, all of whom presented with progressive weakness of both lower limbs; three exhibited autonomic nerve function impairment, such as constipation and dysuria; and one patient presented with polycranial neuritis and Miller–Fisher syndrome, including bilateral facial palsy, dysphagia, diplopia, and ataxia. The nerve conduction velocity and F-wave were abnormal in three patients, and motor conduction pathways were abnormal in one patient. Anti-ganglioside antibodies were tested in three patients (patient 1, patient 2 and patient 3), and GD 1a-IgG was detected in one patient. After treatment, four patients regained the ability to walk independently. No patients were admitted to the ICU, and they were discharged after 12–18 days of hospitalization.

Previous cases of SARS-CoV-2-positive CSF in COVID-19 patients, especially SARS-CoV-2-associated GBS, are rare (10–12). To our knowledge, no cases of SARS-CoV-2-associated GBS with SARS-CoV-2-positive CSF have been previously reported in China. SARS-CoV-2 RNA was detected in the CSF of all four patients described in this study. This may be related to the more advanced and sensitive mNGS technology we used to detect RNA. In previous studies, PCR or virus-specific antibody detection has been used to detect SARS-CoV-2 in CSF. Detection of viruses by PCR is not 100% sensitive because of genetic variations in the virus or technical factors (10). A negative PCR result alone does not exclude nervous system infections (12). The mNGS technique is a high-throughput sequencing technology for nucleic acid detection in clinical samples that does not rely on pathogen culture. It can rapidly and extensively evaluate pathogens by detecting a single sample. Compared to PCR techniques, which can only target specific types of microorganisms, mNGS can detect a wider spectrum of pathogens with lower detection limits. A research reported that mNGS identified more bacteria, viruses, fungi, and mycobacteria than the traditional detection methods (PCR and culture) (13). In recent years, mNGS has shown obvious advantages in virus detection (14, 15); in particular, SARS-Cov-2 was detected for the first time using mNGS technology (16).

The detection rate of SARS-CoV-2 in the CSF largely depends on the disease type and sample collection time (17). Owing to the autoclearance capability of the CSF system, viral titers in the CSF may change during the course of a patient ‘s disease. Therefore, CSF testing may not provide a positive result if sampling is delayed (10). Some autopsy studies have shown that for patients with negative CSF SARS-CoV-2, test results cannot completely exclude the possibility of CNS infection (18, 19). The production of virus-specific antibodies takes time; therefore, antibodies may not be detected at different times.

The differences in the amount of SARS-CoV-2 RNA sequences in the blood and CSF, as well as the differences in clinical manifestations described in this paper, suggest that SARS-CoV-2 may damage the nervous system through different mechanisms. The current results suggest that SARS-CoV-2 may invade the nervous system via direct infection and cause peripheral nerve damage. Previous studies have shown that SARS-CoV-2 can enter the brain through the olfactory bulb and retrograde nerves, and the virus can also enter through sensory fibers of the glossopharyngeal nerve (cranial nerve IX) and vagus nerve (cranial nerve X) (20, 21). We observed that the number of SARS-CoV-2 RNA sequences present in Patient 1 in the initial mNGS assay was as high as 446 in the CSF but only 2 in blood; moreover, Patient 4 showed 371 SARS-CoV-2 RNA sequences in the CSF but not none were detected in blood. Notably, Patient 4 had symptoms of neurological impairment at an earlier time (4 days) from SARS-CoV-2 infection. The patient’s clinical symptoms and electrophysiological findings were consistent with GBS, but her CSF examination 19 days after the onset of peripheral nerve damage symptoms did not show protein-cell separation. At this time, the patient’s multiple indicators of inflammation, including procalcitonin and ferritin in the blood and IgG and IgM in the CSF, were not elevated, the SARS-CoV-2-specific antibody test in the CSF was negative, and only the SARS-CoV-2 IgG antibody test in the blood was positive. When the patient’s blood was retested 10 days later, procalcitonin, ferritin, and IgG levels were elevated. This suggests that the inflammatory response was not significant when symptoms of peripheral nerve damage began to develop and that the peripheral nerve damage may have been directly caused by infection with SARS-CoV-2. It has been found that the presence of SARS-CoV-2 in the brain is not related to the severity of neuroimmune activation, which may be consistent with the characteristics of neurotropic viruses, because they can hide in neurons and are not monitored by the immune system (3, 22). Thus, unless the originally infected neurons are significantly damaged, the immune response in the area of infection will not be effectively activated (23).

SARS-CoV-2 may also invade both the blood and CSF. Patient 2 showed more sequences of SARS-CoV-2 in both the blood and CSF, indicating that SARS-CoV-2 may invade both the blood and CSF and may also invade the nervous system through the blood-brain barrier. The patient had a more rapidly progressive disease course (progression to inability to stand within 3 days), higher CSF protein levels, and more pronounced protein-cell segregation. In addition, the patient had a more significant increase in blood IgG and CSF IgG levels than did the other patients, indicating a more pronounced inflammatory response. Presumably, the neurological damage in this patient may have been caused by two mechanisms: a direct attack of the virus and immune mediation. The inflammatory response may be caused directly by SARS-CoV-2 infection or may be caused by SARS-CoV-2 infection through other autoimmune mechanisms.

SARS-CoV-2 may also induce the production of anti-ganglioside antibodies. Patient 3 had more SARS-CoV-2 RNA sequences in the blood and fewer in the CSF. In addition to the symptoms of peripheral nerve damage, the patient also had meningeal irritation symptoms, such as neck resistance at admission. Enhanced head MRI showed a high FLAIR signal in the bilateral frontotemporal parietal cortex, line-like enhancement in some sulci, and GD1a-IgG antibodies in the blood. It was speculated that the mechanism might be as follows: (1) SARS-CoV-2 in the blood induces the production of GD1a-IgG antibody and leads to peripheral nerve damage and (2) SARS-CoV-2 may enter the brain with blood cells and cause brain tissue damage.

The current results suggest that SARS-CoV-2 may be present in the CSF and blood of some patients with SARS-CoV-2-associated GBS for whom antiviral therapy may be necessary and effective. To date, intravenous immunoglobulin and plasmapheresis are the only immunotherapeutic agents recognized to accelerate recovery from Guillain-Barre syndrome (24). Both treatments have been shown to be equally effective in improving disease outcomes by accelerating recovery, but not in stopping disease progression or altering the extent of neurological damage (25). However, the syndrome remains a serious condition. Even with standard immunotherapy, approximately 5% of people die, and up to 20% are unable to walk independently within 1 year of disease onset (25).

All four patients were treated with azvudine immediately after detection of SARS-CoV-2 in their blood or CSF, as we presumed they had SARS-CoV-2 infection at the time. Patients 1, 2, and 3 were re-examined for RNA in blood and CSF using mNGS 9–10 days after anti-viral treatment with azvudine. SARS-CoV-2 RNA was not detected in the CSF or blood of Patients 1 and 2, and it was detected in the blood but not CSF of Patient 3, with the number of sequences detected in the blood decreasing from 685 at admission to 2. Patient 4 refused to have the CSF re-examined because she did not want to undergo another lumbar puncture. These results suggest that the change in the number of SARS-CoV-2 sequences in the CSF may have a suggestive effect on the severity of disease and the therapeutic effect of patients.

Azvudine, the first domestically developed anti-COVID-19 drug in China, has been shown to shorten the time to nucleic acid negative conversion and cure common and severe COVID-19 patients (26, 27). Interim analyses of phase III studies have shown faster symptomatic improvement and a higher proportion of patients with clinical improvement in patients with COVID-19 treated with azvudine compared with matched controls (28). A real-world study found that azvudine therapy showed substantial clinical benefit in hospitalized patients with COVID-19, particularly in those who were treated for more than 5 days from symptom onset (29). The results of another real-world study showed that azvudine reduced rates of disease progression, as well as COVID-19-related hospitalizations, compared with controls. In addition, it can shorten the duration of fever if given within 3 days of symptom onset (30).

The main clinical manifestations reported here in patients with SARS-CoV-2-associated GBS are similar to those previously reported, but the disease was relatively mild, which may be related to the timely administration of azvudine. It was previously reported that SARS-CoV-2-related GBS mainly manifests as limb weakness, hyporeflexia, sensory disturbance, facial paralysis, ataxia, and autonomic dysfunction. Respiratory failure occurred in 18.2–38% of patients, and admission to the ICU was required in 11.8–46% of patients (5–8). Of the four patients presented herein, none displayed respiratory dysfunction, and none were admitted to the ICU. Nevertheless, this study has several limitations. The significant reduction in SARS-CoV-2 sequences in the blood and CSF cannot be confirmed to be the result of azvudine administration, as the clearance function of the CSF system may also be responsible. In addition, all patients were treated with multiple other medications; therefore, it is uncertain whether the observed improvement was related to treatments other than azvudine.

## 4 Conclusions

The present results showed that SARS-CoV-2 RNA can be detected via mNGS in the CSF of some patients with SARS-CoV-2-associated GBS, suggesting that SARS-CoV-2-associated GBS may have multiple pathogeneses. Furthermore, this study provides a more sensitive method for detecting SARS-CoV-2 and evaluating the treatment effects of SARS-CoV-2-related GBS as well as providing a new basis for further study of the treatment scheme of SARS-CoV-2-related GBS.

## Data availability statement

The datasets presented in this study can be found in online repositories. The names of the repository/repositories and accession number(s) can be found in the article/**Supplementary Material**.

## Ethics statement

The study was reviewed and approved by the Board of Ethics at Tianjin Huanhu Hospital. Written informed consent was obtained from the patients for the publication of any potentially identifiable images or data included in this article.

## Author contributions

YG, CY, and QS contributed to the conception and design of the study. YG, YF, and PW contributed to the administrative support of the study. WZ, HW, and XQ contributed to the provision of study materials for patients. HW and XQ contributed to the collection and assembly of data for the study. YG, CY, and QW contributed to the data analysis and interpretation of the study. All authors contributed to the article and approved the submitted version.
