# Supplementary material for: Case Report: Four cases of SARS-CoV-2-associated Guillain-Barré Syndrome with SARS-CoV-2-positive cerebrospinal fluid detected by metagenomic next-generation sequencing: a retrospective case series from China
Source: Front Immunol. 2023 Aug 28;14:1258579. doi: 10.3389/fimmu.2023.1258579 (PMC10493301; doi:10.3389/fimmu.2023.1258579)
Supplement: Supplementary file 1 [file DataSheet_1.pdf]

1. **Metagenomics next-generation sequencing method is described below:**

1.1 CSF specimens and blood specimens (Patient 1 (Initial examination), Patient 2, Patient 3 and Patient 4) were sent to Tianjin Novogene Medical Laboratory for metagenomic next-generation sequencing (mNGS). RNA was extracted using the Novogene PD-seq^{TM} metagenomic RNA extraction kit (PT105, Tianjin Novogene Medical, Tianjin, China). RNA libraires were constructed using Novogene PD-seq^{TM} metagenomic RNA library kit (PT106, Tianjin Novogene Medical, Tianjin, China). After the removal of rRNA, RNA fragments of size 250–300 bp were obtained using the enzyme. The first strand cDNA was synthesized, and then the second strand cDNA was synthesized, followed by terminal repair and A-tailing reactions. Adapters were ligated onto the A-tailed fragments. Fragments with adapters were purified and amplified using PCR. After purifying the PCR product, the libraries were pooled and sequenced for 50 bp single ends on an GenoLab M (GeneMind Biosciences Co., Ltd.，Shenzhen，China) machine. The data output of each sample was guaranteed to be more than 30M reads. Nuclease free water and plasma from a non-infected individual were used as negative controls to monitor contamination throughout the workflow. Adapters and the reads of low quality or length < 15 bp were filtered out using fastp. Bowtie2 (version 2.3.5.1) was used to map human reference genomes (GRCh38+YHref) to identify and remove the human sequencing data. After removing human reads, the remaining reads were aligned with our curated microbial genome database (PD-seqTM database version 1.0), whose genome sequences were derived from the RefSeq, GenBank, and NT databases. The database used in this study contained 9,295 bacterial species, 7,210 viral species, 412 fungal species, 104 parasites. We identified the species by mapping our curated microbial database using the Kraken2+Bracken workflow. Kraken2 (version2.1.2) was used to annotate the sequence reads.

1.2 The CSF specimen and blood specimen (Patient1 (Re-examination)) were sent to V-Medical Laboratory Co., Ltd. for mNGS. The mNGS test was conducted with the use of a protocol for the validated mNGS assay following described. Total RNA was extracted using the TIANamp Virus RNA Kit (DP315-R, Tiangen Biotech, Beijing, China), a commercial kit. Subsequently, libraries were constructed using the Nextera XT DNA Library Preparation Kit (Illumina) and sequenced on the Illumina NextSeq 550 platform. Same protocol was performed for negative control and positive control simultaneously. High-quality sequencing data were generated by removing low-quality reads, followed by computational subtraction of human host sequences mapped to the human reference genome (hg19) using Burrows-Wheeler Alignment. The remaining data which removed low-complexity reads were classified by aligning to the NCBI microbial genome database (ftp://ftp.ncbi.nlm.nih.gov/genomes/) which contains genome sequences for about 29844 pathogens.

2. Sensitivity and specificity of mNGS

In the cases of nervous system infection, the proportion of mNGS positive with traditional etiological methods was 22.5%-52.6%, and the sensitivity of cerebrospinal fluid mNGS in the diagnosis of encephalitis and meningitis was 73% and the specificity was 99% (1-6).

Reference

1. Xing XW, Zhang JT, Ma YB, He MW, Yao GE, Wang W, et al. Metagenomic Next-Generation Sequencing for Diagnosis of Infectious Encephalitis and Meningitis: A Large, Prospective Case Series of 213 Patients. *Front Cell Infect Microbiol* (2020). 10:88. doi: 10.3389/fcimb.2020.00088
2. Liu L, Zhang JX, Di XM, et al. Discussion on objective inclusion criteria for sending cerebrospinal fluid pathogen metagenomic next-generation sequencing. Chin J Contemp Neurol Neurosurg (2021), 21(5): 350-357
3. Zhang Y, Cui P, Zhang HC, Wu HL, Ye MZ, Zhu YM, Ai JW, Zhang WH. Clinical application and evaluation of metagenomic next-generation sequencing in suspected adult central nervous system infection. J Transl Med (2020). 18(1):199. doi: 10.1186/s12967-020-02360-6
4. Fan S, Wang X, Hu Y, et al. Metagenomic next-generation sequencing of cerebrospinal fluid for the diagnosis of central nervous system infections: a multicentre prospective study[EB/OL]. (2019 - 06 - 10) [2020 - 09 - 17].https://www.biorxiv.org/content/10.1101/658047v1.
5. Wilson MR, Sample HA, Zorn KC, Arevalo S, Yu G, Neuhaus J, et al. Clinical Metagenomic Sequencing for Diagnosis of Meningitis and Encephalitis. N Engl J Med (2019). 380(24):2327-2340. doi: 10.1056/NEJMoa1803396
6. Miller S, Naccache SN, Samayoa E, Messacar K, Arevalo S, Federman S, et al. Laboratory validation of a clinical metagenomic sequencing assay for pathogen detection in cerebrospinal fluid. Genome Res (2019). 29(5):831-842. doi: 10.1101/gr.238170.118

3.The exact antibody type is described below：

Patients 1, 2, and 3 were tested for autoimmune encephalitis, central nervous system demyelination, ganglioside antibodies, Ranvier node antibodies, and paraneoplastic antibodies in the blood and CSF. Patient 4 refused antibody screening. Patients 1 and 2 tested negative for all the aforementioned antibodies; Patient 3 tested positive for GD1a-IgG and negative for the other antibodies.

Autoimmune encephalitis antibodies included antibodies against NMDAR, AMPAR1, AMPAR2, LGI1, CASPR2, GABABR, DPPX, IgLON5, GlyR1, D2R, GAD65, mGluR5, mGluR 1, GABAARα1, GABAARβ3, neurexin3α, MOG, and GFAP. Demyelinating antibodies in the CNS included antibodies against AQP4, MOG, MBP, GFAP, AQP1, and flotilin-1/2. Anti-ganglioside antibodies included antibodies against GM1-IgM, GM1-IgG, GM2-IgM, GM2-IgG, GM3-IgM, GM3-IgG, GM4-IgM, GM4-IgG, GD1a-IgM, GD 1a-IgG, GD 1b-IgM, GD1b-IgG, GD 2-IgM, GD2-IgG, GD3-IgM, GD3-IgG, GQ 1b-IgM, GQ1b-IgG, GT 1a-IgM, GT 1b-IgM, GT 1b-IgG, sulfatides-IgM, sulfatides-IgG, and MAG-IgM. Nodal and paranodal antibodies included antibodies against NF155-IgG, NF186-IgG, CNTN1-IgG, CNTN2-IgG, and CASPR1-IgG. Paraneoplastic antibodies included antibodies against Hu, Yo, Ri, CV2, amphiphysin, Ma1, Ma2, SOX1, Tr, Zic4, titin, recoverin, PKCγ, and GAD65.

4. The datasets are presented in an online repository

The raw sequence data reported in this paper have been deposited in the Genome Sequence Archive (Genomics, Proteomics & Bioinformatics 2021) in National Genomics Data Center (Nucleic Acids Res 2022), China National Center for Bioinformation / Beijing Institute of Genomics, Chinese Academy of Sciences (GSA: CRA012180) that are publicly accessible at https://ngdc.cncb.ac.cn/gsa.
